# Editorial for the SEB Florence special issue: functional organisation of the nuclear periphery

**DOI:** 10.1080/19491034.2019.1643140

**Published:** 2019-07-25

**Authors:** David E Evans, Katja Graumann, Roland Foisner

**Affiliations:** a Department of Biological and Medical Sciences, Faculty of Health and Life Sciences, Oxford Brookes University, Oxford UK; b Max Perutz Laboratories, Medical University of Vienna, Vienna, Austria

This special issue comprises papers based in a meeting of the Nuclear Dynamics Special Interest Group of the Society for Experimental Biology. The session was entitled ‘Functional organisation of the nuclear periphery’ and was held at the Society’s Annual Main Meeting in the beautiful city of Firenze (Florence) in July 2018. Organized by Katja Graumann and David Evans from Oxford Brookes University, Oxford UK and Roland Foisner, Medical University Vienna, Austria, the session highlighted novel research in plant, animal and fungal nuclear biology since the previous meeting of the group in Brighton, UK in 2016[1].

The meeting was supported by attendance of members of the INDEPTH (Impact of Nuclear Domains On Gene Expression and Plant Traits) COST Action CA16212 (https://www.cost.eu/actions/CA16212/). A review article in this special issue of *Nucleus* by members of Workgroup 1 of the COST Action provides insights into microscopy methods for studying 3D nuclear architecture in plants and accompanying challenges being addressed in the consortium[2]. Other papers highlight progress in understanding mechanisms for the dynamic organisation of the nuclear periphery, both in interphase and in dividing cells, including the role of lamina-associated domains (LADs) in spatial chromatin organization[3], the role of the nucleolus in anchoring chromatin structures[4], the contribution of structural maintenance of chromatin (SMC) complexes to chromatin organisation[5], and the function of plant KASH domain proteins in attaching the nucleus to the actin cystoskeleton[6]. The structural role of the nuclear envelope in plant mitosis and meiosis is reviewed [7] with a further paper presenting evidence for the role of lamins in meiotic chromosome movements at the nuclear periphery in *C. elegans*[8]. The final paper in the special issue [9] provides a comprehensive exploration of the plant nuclear proteome using a sequential extraction method which has significantly increased the number of plant nuclear proteins identified.

As editors, we are grateful to all the contributors, both to the meeting and to this special issue. Their enthusiasm and willingness to share their insights and expertise with others was reflected in their presentations and is evident in the resulting papers. The cross-kingdom format of these meetings provides a fertile environment for generating research ideas and for development of collaborations and this is evidenced in the progress made by the members of the Group.
